# Assumptions and analysis planning in studies with missing data in multiple variables: moving beyond the MCAR/MAR/MNAR classification

**DOI:** 10.1093/ije/dyad008

**Published:** 2023-02-13

**Authors:** Katherine J Lee, John B Carlin, Julie A Simpson, Margarita Moreno-Betancur

**Affiliations:** Clinical Epidemiology and Biostatistics Unit, Murdoch Children’s Research Institute, Melbourne, Australia; Department of Paediatrics, University of Melbourne, Australia; Clinical Epidemiology and Biostatistics Unit, Murdoch Children’s Research Institute, Melbourne, Australia; Department of Paediatrics, University of Melbourne, Australia; Centre for Epidemiology and Biostatistics, Melbourne School of Population and Global Health, University of Melbourne, Melbourne, VIC, Australia; Centre for Epidemiology and Biostatistics, Melbourne School of Population and Global Health, University of Melbourne, Melbourne, VIC, Australia; Clinical Epidemiology and Biostatistics Unit, Murdoch Children’s Research Institute, Melbourne, Australia; Department of Paediatrics, University of Melbourne, Australia

**Keywords:** Missing data, missing at random, missing not at random, multiple imputation, complete records analysis, sensitivity analysis, directed acyclic graphs, recoverability

## Abstract

Researchers faced with incomplete data are encouraged to consider whether their data are ‘missing completely at random’ (MCAR), ‘missing at random’ (MAR) or ‘missing not at random’ (MNAR) when planning their analysis. However, there are two major problems with this classification as originally defined by Rubin in the 1970s. First, when there are missing data in multiple variables, the plausibility of the MAR assumption is difficult to assess using substantive knowledge and is more stringent than is generally appreciated. Second, although MCAR and MAR are sufficient conditions for consistent estimation with specific methods, they are not necessary conditions and therefore this categorization does not directly determine the best approach for handling the missing data in an analysis. How best to handle missing data depends on the assumed causal relationships between variables and their missingness, and what these relationships imply in terms of the ‘recoverability’ of the target estimand (the population parameter that encodes the answer to the underlying research question). Recoverability is defined as whether the estimand can be consistently estimated from the patterns and associations in the observed data without needing to invoke external information on the extent to which the distribution of missing values might differ from that of observed values. In this manuscript we outline an approach for deciding which method to use to handle multivariable missing data in an analysis, using directed acyclic graphs to depict missingness assumptions and determining the implications in terms of recoverability of the target estimand.

Key Messages

- Whether incomplete data are ‘missing completely at random’, ‘missing at random’ or ‘missing not at random’ is not easy to assess substantively with multivariable missingness, nor does this categorization correspond to necessary conditions for consistent estimation with specific methods; hence it does not directly determine the best method of handling missing data in an epidemiological analysis, as commonly implied.
- Directed acyclic graphs (DAGs) that include nodes to indicate missingness for each incomplete variable, referred to as m-DAGs, provide a powerful alternative approach to depicting and assessing assumptions about the causes of missing data.
- From these assumptions, we can in principle determine whether and how a parameter of interest may be ‘recovered’, i.e. consistently estimated from the patterns and associations in the observed data, which can be used to guide the choice of missingness method.
- Analysts are encouraged to approach problems with missing data in multiple variables by developing an m-DAG for the epidemiological analysis and considering recoverability to guide the missingness method, rather than trying to assess and rely on the validity of a missing at random assumption.

## Introduction

Missing data arise in almost all research studies. In planning analyses, researchers faced with incomplete data are encouraged to consider whether their data are ‘missing completely at random’ (MCAR), ‘missing at random’ (MAR) or ‘missing not at random’ (MNAR).^1^ Informally, these are understood as follows: MCAR as the assumption that missingness does not depend on observed or missing data; MAR (but not MCAR) as the assumption that missing data are unrelated to unobserved values given the observed data; and MNAR as the negation of MAR, arising if missingness is related to unobserved values given the observed data. This classification is used to help plan the analytic approach for handling missing data.

There are two major problems with this MCAR/MAR/MNAR classification. First, although the above interpretations are correct when there is missingness in a single variable, this is not the case when there are multiple incomplete variables.^2^^,^^3^ With multivariable missingness, there can be different patterns of missingness within a data set (referring to which variables are missing together). The missingness assumptions relate to the probability distribution of missingness patterns and how this relates to observed and unobserved values in that pattern. Seaman *et al*.^4^ clarified the definition of MAR and its connection with inference under various paradigms, but the precise definition is not widely understood and its plausibility is not easily assessed.

Second, even if these terms were clearly understood and readily applied, this categorization does not provide a direct guide to how the missing data should be handled in a given analysis. For example, under the assumption that data are MCAR, although it is well known that restricting the analysis to observations with complete data, a so-called complete records analysis (CRA), will result in consistent estimation,^5^ there may be more efficient approaches that use information from variables that are not in the target analysis (termed auxiliary variables).^6^ Likewise, if data are assumed to be MAR, this does not imply that an approach that models the dependency of the missingness on the observed data, such as multiple imputation (MI), is required for consistent estimation—a CRA could also be consistent for the estimand of interest. An example is when estimating the effect of an exposure on an outcome and the missing data for any variable do not depend on the outcome values,^6^^,^^7^ as we explain further below. Finally, if it is believed that data are MNAR, this does not necessarily imply that an approach allowing for missing values to follow a distribution that is different from that of the observed data is required.^8^

**Figure 1:**
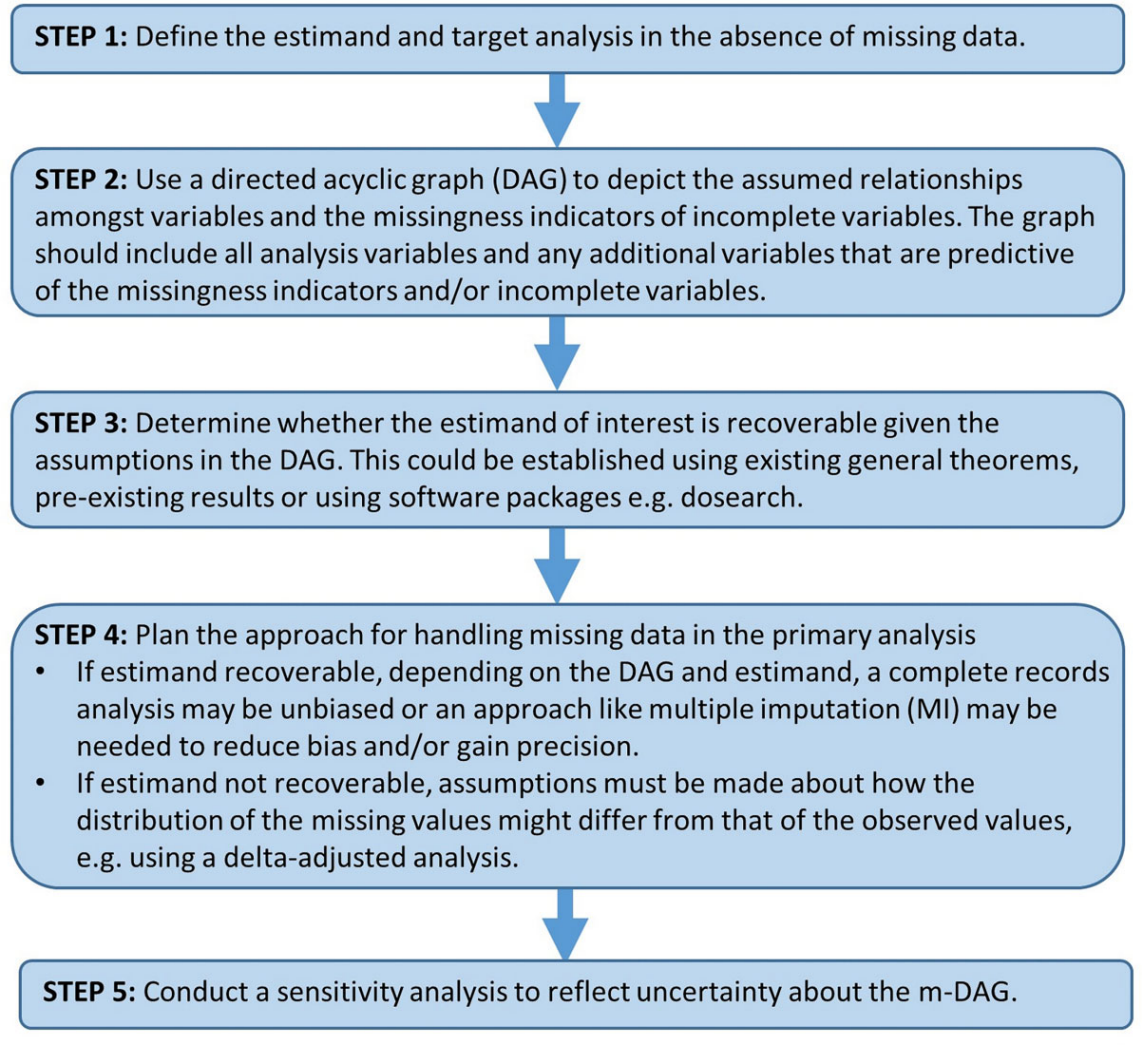
The process of analysis planning when there are multiple variables with incomplete data

How best to handle missing data in an analysis depends on the assumed causal relationships between the variables in the data set and their missingness, and what these imply in terms of the ‘recoverability’ of the estimand.^7^^,^^9^ In this manuscript we outline a general approach to deciding how best to handle missing data in an analysis based on the concept of recoverability. We focus on the context in which there are multiple incomplete variables (where the MCAR/MAR/MNAR classification is problematic), although the same concepts can be useful with univariable missingness. The steps of this approach are summarized in Figure 1.

## Illustrative example

We revisit an example of a point-exposure study from the Longitudinal Study of Australian Children.^10^ The analysis aimed to estimate the effect of maternal mental illness at age 4–5 years (15% missing out of *n* = 4882) on child behaviour (measured by using the Strengths and Difficulties Questionnaire, SDQ [range 0–40]) at age 8–9 years (23% missing), within the causal framework outlined below, requiring adjustment for a selected set of confounders (19% incomplete).^7^

## The approach

### Step 1: Define the estimand and target analysis in the absence of missing data

As with any analysis, the first step is to define the estimand of interest (the quantity we wish to estimate in our statistical analysis) and how it would be estimated if there were no missing data. In our example, the estimand of interest is the causal effect of exposure to maternal mental illness on child behaviour, which under a set of causal and parametric assumptions, including no effect modification by any confounding variable, is equal to the exposure coefficient in a main-effects linear regression model for the outcome adjusted for potential confounders.

### Step 2: Use a directed acyclic graph to depict the assumed relationships between analysis variables and missingness indicators

Directed acyclic graphs (DAGs) provide a graphical tool for displaying the causal assumptions made in an analysis. The nodes represent the variables in the analysis, or common causes of them, with arrows depicting the assumed relationships between the variables.^11^^,^^12^

Such graphs can be extended to depict assumptions regarding the causes of missing data by including indicators of missingness for each incomplete variable as nodes, as well as variables that are predictive of the missingness indicators and/or incomplete variables, in the DAG. We refer to these extended graphs as missingness DAGs or m-DAGs, as they are extensions to standard DAGs (common terminology in the epidemiological literature), although they were originally referred to as M-graphs in the literature.^5^^,^^9^^,^^13^ As with any DAG, the m-DAG should be drawn by considering the potential presence of each arrow based on context-specific substantive knowledge. If is not clear whether an arrow (causal effect) exists or not, the most conservative approach is to include the arrow in the m-DAG.

**Figure 2:**
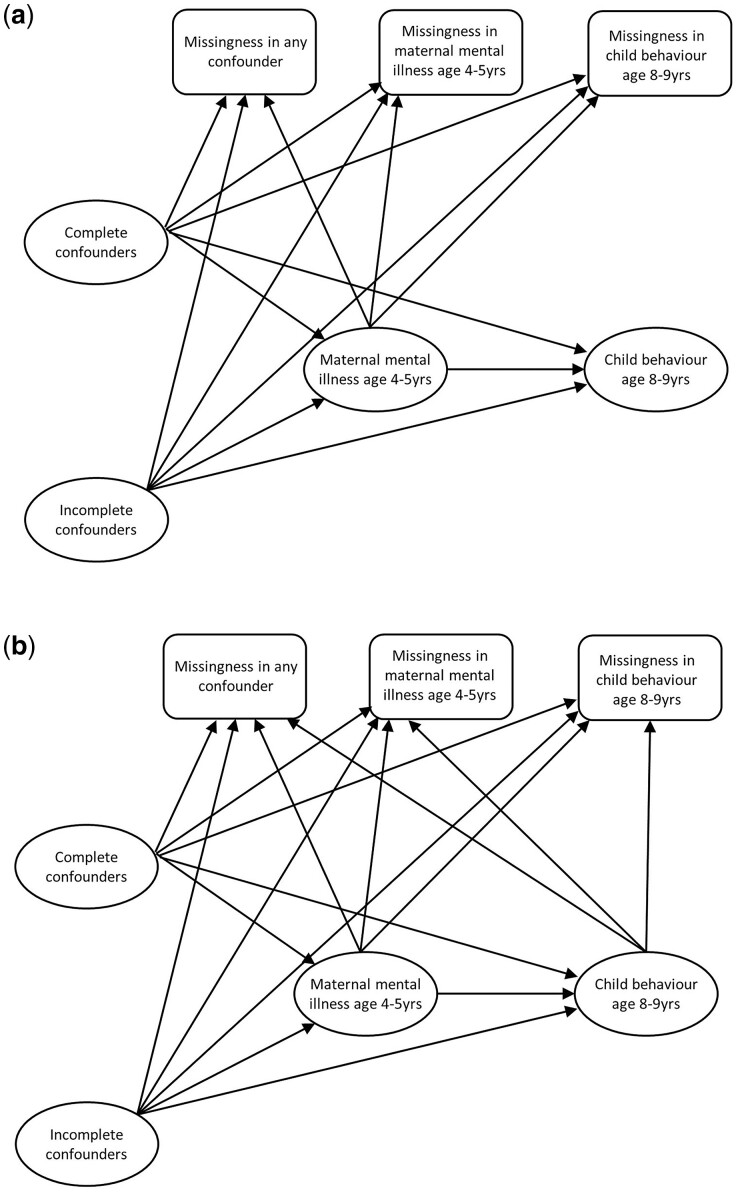
Two potential missingness Directed Acyclic Graphs (m-DAGs) for the effect of maternal mental illness at age 4–5 years on child behaviour at age 8–9 years in the Longitudinal Study of Australian Children case study. Note, in this figure we collapse the complete confounders into a single node, and the incomplete confounders into a single node, because the causal relationships amongst the collapsed variables, or their individual relationships with other variables in the diagram, do not affect the findings regarding recoverability. Based on the literature, there was likely a causal relationship between the incomplete confounders (i.e. maternal alcohol drinking and smoking and child physical functioning) and missingness hence the inclusion of the arrow between the node representing the incomplete confounders and the three missingness indicators. It was also likely that maternal mental illness (the exposure) was associated with missingness hence the inclusion of an arrow between the exposure the three missingness indicators. In (a) we assume it was unlikely that there would be an association between child behaviour at age 8–9 years (the outcome) and missingness, so there is no arrow between the outcome and the three missingness indicators. In (b) we assume that the outcome was associated with missingness and hence include an additional arrow between the outcome and each of the three missingness indicators. See Moreno-Betancur *et al.*^7^ for more details

Figure 2 shows two possible m-DAGs for the example, with arrows representing the assumed causal relationships amongst analysis variables and missingness indicators based on the literature and subject-matter experts’ views. In these diagrams, we considered a single indicator of missingness in any of the confounders—a simplification that facilitates the elicitation of expert knowledge in the context of multiple incomplete confounders while still capturing the detail relevant for most practical purposes.^7^ Furthermore, the set of complete confounders and the set of incomplete confounders have each been collapsed into a single node for conciseness, since neither the assumed causal relationships amongst variables within these sets nor their individual relationships with other nodes in the diagram have a material effect on the following steps, i.e. on the recoverability of parameters and implied approach to handle missing data.

### Step 3: Determine whether the estimand of interest is recoverable

There are graphical rules that allow determination of whether variables are independent or conditionally independent given other variables in the DAG.^12^^,^^13^ These conditional independencies can in turn be used to determine mathematically whether the estimand of interest, which relates to the true (infinite-sample) distribution of the data if it were complete, can be expressed as a function of the true distribution of the incomplete data. If so, the parameter is ‘recoverable’, which implies it can be consistently estimated from the patterns and associations in the observed data without needing to invoke external information on the extent to which the distribution of missing values differs from that of observed values.^9^^,^^13^

Determining recoverability is not straightforward; it requires complex mathematical derivations and is specific to the estimand and the m-DAG of interest. In the Supplementary material (available as Supplementary data at *IJE* online) we describe the process for determining recoverability in our case study in which we wish to estimate an exposure effect using outcome regression to adjust for confounding under an expanded version of the m-DAG in Figure 2a (where there may be unmeasured common causes of the confounders and of the missingness indicators).

Mohan *et al.*^5^^,^^9^^,^^14^^,^^15^ have conducted extensive theoretical work, resulting in theorems that establish (non-)recoverability for certain estimands in general m-DAGs satisfying various conditions, with extensions to settings where it may be possible to leverage information from other variables and features of the data.^5^^,^^16^ Although useful, these results do not cover every possible m-DAG and target estimand, and their application to determine recoverability of a given estimand under a specific m-DAG may require further mathematical considerations and derivations.

In previous work, with the aid of Mohan *et al*.’s theoretical results, we have conducted such an analysis, examining recoverability of specific estimands in a range of possible m-DAGs in epidemiological studies. Specifically, Moreno-Betancur *et al*.^7^ determined the recoverability of three key estimands, the marginal mean of the exposure and outcome, and the regression-adjusted association, in 10 m-DAGs for a point-exposure study with incomplete exposure, outcome and confounders. These m-DAGs are ‘canonical’ in that they each represent a set of ‘nested’ m-DAGs that may be obtained by removing arrows and for which the same recoverability results apply, and they cover all possible m-DAGs with distinct recoverability properties in a point-exposure study under a set of overarching assumptions, namely that there are (i) no unmeasured common causes of a variable and a missingness indicator in the m-DAG, (ii) no measured common causes of a variable and a missingness indicator in the m-DAG that are not included in the m-DAG, and (iii) no direct arrows from missingness indicators to other variables, or to other missingness indicators.^7^ Hence, a simple way to determine recoverability in a point-exposure study in which these overarching assumptions are met is to use these pre-existing results. We illustrate this approach in our case study below. The disadvantage of using these pre-existing results is that they are limited to certain parameters in point-exposure studies and the approach cannot be used if the overarching assumptions underlying the canonical m-DAGs are not deemed plausible, e.g. if there are auxiliary variables (predictors of missing values not in the target analysis) that are also predictors of missingness indicators.

Another way to determine recoverability is to use the dosearch package in R,^17^ which uses do-calculus and standard probability manipulations using a search-based algorithm to provide recoverability results.^18^ Although reasonably comprehensive and easy to use, this algorithm is not ‘complete’ in that it cannot solve all problems.^19^

In our case study, the primary m-DAG displaying appropriate assumptions for our example (Figure 2a) satisfies the overarching assumptions for the canonical m-DAGs in Moreno-Betancur *et al.*,^7^ making it possible to determine recoverability directly from those results. To apply this approach, the researcher should start with an m-DAG with all key arrows, i.e. arrows from each incomplete variable to each missingness indicator (called m-DAG J in that paper), and for each arrow determine whether there is any equivalent arrow in their study m-DAG. If not, then drop the arrow. Once each arrow has been considered, the simplest canonical m-DAG with all the remaining arrows should be selected and recoverability or otherwise of the estimand determined from the results in the paper. If there are multiple m-DAGs that align with the study m-DAG and the parameter is recoverable in one of them, then the parameter is recoverable in the study**.** The study m-DAG in Figure 2a can be mapped to m-DAG type E, under which the exposure effect estimand is recoverable.

### Step 4: Plan the approach for handling missing data in the primary analysis

If in Step 3 the parameter is determined to be recoverable, it can be consistently estimated without external information on how the distribution of missing values may differ to that of the observed values. The questions are then whether a CRA will be unbiased and whether standard MI (or an alternative approach that models the dependency of the missingness on the observed data) would offer reduced bias and/or improved precision. The process of determining recoverability may suggest what analytic method could be used from the expression linking the target parameter to the observable data. For example, it can show whether a CRA approach will be unbiased, as is the case in the example (see Supplementary material available as Supplementary data at *IJE* online). If auxiliary variables were required to establish recoverability, the CRA is likely to be biased and MI including the auxiliary variables should reduce bias. Additionally, if auxiliary variables are available (even if not used to establish recoverability) they can be used to gain precision by incorporating them in the MI procedure.

If the parameter is not recoverable, assumptions must be made about how the distribution of the missing values might differ from that of the observed values. This can be achieved using either a selection model or a pattern mixture modelling approach.^20^ The latter is more easily interpretable and can be implemented within the MI framework. Using this approach, the distributional differences between observed and unobserved values of a variable are specified in the imputation model by including a term representing the missingness indicator of the variable being imputed as a predictor. The coefficients of these terms are often referred to as ‘delta(s)’.^21^ By definition, the delta(s) cannot be estimated from the data so need to be specified based on content expert knowledge and/or available literature. Such an analysis is often called an ‘MNAR analysis’; we prefer ‘delta-adjusted analysis’.

In the case study, under the primary m-DAG (Figure 2a), the estimand is recoverable and per the derivation in the Supplementary material (available as Supplementary data at *IJE* online) (or pre-existing results for the canonical m-DAGs) the CRA analysis would be consistent. Since there are no auxiliary variables, a CRA would be the preferred analysis. Table 1 presents the results from the CRA for this case study, where we see an estimated difference of just over 0.5 on the SDQ score (meaning slightly poorer child behaviour) between those exposed to maternal mental illness compared with those unexposed, albeit with substantial uncertainty.

**Table 1:** Results from the case study assessing the causal effect of maternal mental illness on child behaviour in the Longitudinal Study of Australian Children

Method for handling missing data	Estimate (95% CI)
Primary: complete records analysis	0.59 (0.20, 0.98)
Sensitivity: delta-adjusted analysis	
- delta^a^ = 0.56 (lower quartile)	0.68 (0.26, 1.10)
- delta^a^ = 2.12 (median)	0.76 (0.32, 1.18)
- delta^a^ = 4.92 (upper quartile)	0.9 (0.44, 1.35)

Results represent the mean difference (and its 95% confidence interval [CI]) in the child behaviour measure (Strengths and Difficulties Questionnaire [SDQ]) for those exposed to maternal mental illness compared with those unexposed, estimated using a linear regression of child behaviour on maternal mental illness and a set of potential confounders.

a Average difference in the mean SDQ score for those with and without missing data on the SDQ, conditional on all analysis variables, as obtained from elicitation for the distribution of the marginal equivalent of this value from content experts.

### Step 5: Conduct a sensitivity analysis to reflect uncertainty about the m-DAG

The final step is to consider whether there is uncertainty regarding the assumed m-DAG. If there is, then a sensitivity analysis should be conducted under alternative plausible m-DAGs. The results from these analyses should be presented along with those from the primary analysis.^22^ In our example, there was uncertainty regarding whether the missingness in the incomplete variables was related to the outcome (Figure 2b). This new m-DAG maps to m-DAG J from Moreno-Betancur *et al.*^7^ and hence the regression coefficient is not recoverable, so a delta-adjusted analysis would be required. Table 1 also presents the results from a delta-adjusted analysis for the case study, conducted using MI via not at random fully conditional specification,^23^ applying a delta adjustment to the imputed values for the missing outcome. The values of delta (the average difference in the mean SDQ score for those with and without missing data on the SDQ, conditional on all analysis variables) were selected to be 0.56, 2.12 and 4.92, the lower quartile, median and upper quartile for the distribution of the marginal equivalent of this value based on elicitation from content experts.^24^ These analyses resulted in slightly larger, although still small, estimated differences in behavioural problems for those exposed to maternal mental illness compared with those unexposed.

## Discussion

We outline an approach for deciding the best method of analysis in the presence of multiple incomplete variables that focuses on whether, given missingness assumptions, a parameter is recoverable, i.e. whether it can be consistently estimated from the patterns and associations in the observed data alone. Importantly, our approach avoids the need to categorize multivariable incomplete data as MCAR, MAR or MNAR. Instead, we use m-DAGs to depict and assess the analyst’s assumptions regarding the causes of missingness in each incomplete variable. Then the task is to determine recoverability given assumptions in the m-DAG, which can be used to guide the choice of analytic approach.

By focusing on the key concept of recoverability, the proposed approach informs researchers as to whether a delta-adjusted analysis is required. If so, then external information is needed on the extent to which the distribution of missing values might differ from that of observed values, ideally considering a range of values to reflect the uncertainty. In practice, delta-adjusted analyses are rarely conducted due to the difficulty of elicitation of judgements about likely differences and the need for more sophisticated coding, with authors simply stating that data were assumed to be MAR without justification. Our approach may help to change this practice, which is at odds with recommendations to consider the plausibility of assumptions and plan analyses accordingly.^25^

The difficult step in the proposed approach is determining recoverability, and although there are several available approaches outlined here, none is entirely satisfactory. We illustrated one approach using pre-existing results that are readily applicable in the context of a point-exposure study, albeit for a limited set of parameters and in the absence of auxiliary variables that are also predictors of missingness. For other parameters in other scenarios or study types, recoverability would need to be determined on a case-by-case basis, e.g. through direct mathematical derivation or using dosearch in R. How to further facilitate this process for practical applications is an area requiring further investigation.

## Ethics approval

The Longitudinal Study of Australian Children received ethics approval from the Australian Institute of Family Studies Ethics Committee for each wave of data collection.

## Supplementary Material

dyad008_Supplementary_Data

## Data Availability

All data used in this manuscript are available from the Longitudinal Study of Australian Children (https://dataverse.ada.edu.au/dataverse/lsac).
