# Supplementary material for: Assumptions and analysis planning in studies with missing data in multiple variables: moving beyond the MCAR/MAR/MNAR classification
Source: Int J Epidemiol. 2023 Feb 13;52(4):1268–75. doi: 10.1093/ije/dyad008 (PMC10396404; doi:10.1093/ije/dyad008)
Supplement: dyad008_Supplementary_Data [file dyad008_supplementary_data.docx]

**Supplementary Material: Illustration of how recoverability of a parameter is determined**

Here we provide an illustration of how recoverability of a parameter is determined from the assumptions encoded in an m-DAG, in the context of the example from the main text where the target estimand is the regression-adjusted exposure-outcome association and the primary m-DAG is that in Figure 2a of the manuscript. Interested readers are referred to the tutorial of Thoemmes and Mohan^1^ for further illustrations.

We first introduce some notation. Let $X$ represent the exposure, $Y$ the outcome, $\boldsymbol{Z}_{\boldsymbol{1}}$ and$\boldsymbol{Z}_{\boldsymbol{2}}$_,_ sets of complete and incomplete confounders, respectively, $U$ unmeasured common causes of the confounders, $M_{X}$, $M_{Y}$ and $M_{\boldsymbol{Z}_{\boldsymbol{2}}}$ indicators of missing data in the exposure, outcome and incomplete confounders, and $W$ unmeasured common causes of the missingness indicators.

A schematic representation of the m-DAG of Figure 2a of the main text using this notation is given in the Figure below, with additional inclusion of potential unmeasured common causes of the confounders ($\boldsymbol{U})$ and of the missingness indicators ($\boldsymbol{W}$).


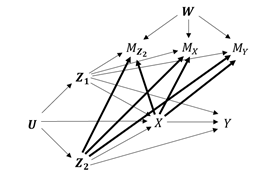


*Figure: Expanded representation of the m-DAG in Figure 2a of the manuscript.*

We now show how one can determine the recoverability of the target estimand (regression-adjusted exposure-outcome association) given the assumptions in the m-DAG in the Figure above.

This process has 2 steps:

## *Determine conditional independencies implied by the m-DAG using d-separation*

The causal assumptions depicted by an m-DAG translate into a specific set of conditional independence properties between the variables through a graphical criterion called d-separation^2^. If two variables are d-separated given a conditioning set of other variables in the graph then they are statistically independent given that conditioning set.

To determine whether two variables are d-separated given a conditioning set, we must examine each path (i.e. sequence of arrows) connecting the two variables and determine whether it is “open” or “closed” after conditioning on the given set. This requires understanding the conditions under which a path is “open” or closed”:

1. Prior to conditioning, a path is open if it does not contain a “collider” (a variable with two arrows pointing towards it)
2. Prior to conditioning, a path is closed if it contains a collider
3. Conditioning on a non-collider closes a path
4. Conditioning on a collider or a descendent of a collider may open a path if it is not closed otherwise (e.g. by another collider not conditioned upon, or by conditioning on a non-collider)

Two variables are d-separated given a conditioning set if after conditioning on all variables of the set simultaneously there remains no open path connecting them.

This definition and the above conditions to determine d-separation apply in the same way whether the graph is a DAG or an m-DAG. However, in missing data problems, the key interest is to determine the d-separation of substantive variables and missingness indicators conditional on other variables. This is because the implied conditional independence properties are what may allow us, in the next step, to express the target parameter in terms of the distribution of the incomplete data, which would mean the parameter is recoverable. Importantly, the specific conditional independence properties between variables and missingness indicators that are useful to determine recoverability depend on both the m-DAG and target parameter. This is why recoverability generally needs to be ascertained mathematically on a case-by-case basis.

As an example, in the m-DAG in the Figure, $Y$ and $M_{Y}$ are d-separated given $\{X,\boldsymbol{Z}_{\boldsymbol{1}}\boldsymbol{,}\boldsymbol{Z}_{\boldsymbol{2}}\}$. This is determined by noting that the all paths between them are closed by conditioning on the variables in this set. For example:

- The paths $M_{Y}\leftarrow X\to Y$ and $M_{Y}\leftarrow W\to M_{X}\leftarrow X\to Y$ are closed by conditioning on the non-collider $X$
- The paths $M_{Y}\leftarrow\boldsymbol{Z}_{\boldsymbol{1}}\to Y$ and $M_{Y}\leftarrow{W\to M_{\boldsymbol{Z}_{\boldsymbol{2}}}\leftarrow\boldsymbol{Z}}_{\boldsymbol{1}}\to Y$ are closed by conditioning on the non-collider $\boldsymbol{Z}_{\boldsymbol{1}}$
- The paths $M_{Y}\leftarrow\boldsymbol{Z}_{\boldsymbol{2}}\to Y$ and $M_{Y}\leftarrow{W\to M_{\boldsymbol{Z}_{\boldsymbol{2}}}\leftarrow\boldsymbol{Z}}_{\boldsymbol{2}}\to Y$ are closed by conditioning on the non-collider $\boldsymbol{Z}_{\boldsymbol{2}}$

Similarly, $Y$ is d-separated from $M_{X}$ and $M_{\boldsymbol{Z}_{\boldsymbol{2}}}$ given $\{X,\boldsymbol{Z}_{\boldsymbol{1}}\boldsymbol{,}\boldsymbol{Z}_{\boldsymbol{2}}\}$.

This implies the following conditional independencies in m-DAG E:

$$(M_{\boldsymbol{Z}_{\boldsymbol{2}}},M_{X},M_{Y})\perp Y | (\boldsymbol{Z}_{\boldsymbol{1}}\boldsymbol{,}\boldsymbol{Z}_{\boldsymbol{2}},X)$$

As seen in the next section, these conditional independencies are useful for determining the recoverability of the target parameter in this example. Of note, ascertaining d-separation is tedious in practice and software such as the R package “dagitty”^3^ can help in this task.

## *Mathematically express the parameter of interest as a function of the distribution of the incomplete data distribution*

The target estimand is the coefficient of the exposure in a generalized linear regression model for $Y$ given$X\boldsymbol{,}\boldsymbol{Z}_{\boldsymbol{2}}\boldsymbol{,}\boldsymbol{Z}_{\boldsymbol{1}}$ (e.g. regression-adjusted mean difference, log-odds ratio, log-risk ratio), which, assuming the exposure ($X)$ is coded 0/1, is given by:

$$g\left\{ E\left( Y|X=1, \boldsymbol{Z}_{\boldsymbol{1}}\boldsymbol{,}\boldsymbol{Z}_{\boldsymbol{2}} \right) \right\}-g\left\{ E\left( Y|X=0, \boldsymbol{Z}_{\boldsymbol{1}}\boldsymbol{,}\boldsymbol{Z}_{\boldsymbol{2}} \right) \right\}$$

where $g$ is the link function in the generalized linear model (e.g. identity for linear regression, logit for logistic regression).

By definition $E\left( Y|X, \boldsymbol{Z}_{\boldsymbol{1}}\boldsymbol{,}\boldsymbol{Z}_{\boldsymbol{2}} \right)$ is a function of the conditional distribution $P\left( Y=y|X, \boldsymbol{Z}_{\boldsymbol{1}}\boldsymbol{,}\boldsymbol{Z}_{\boldsymbol{2}} \right)$. In order for a distribution (and therefore a function of the distribution) to be recoverable, it suffices to show that the distribution can be expressed as a function of the incomplete data distribution, which follows directly from the conditional independence derived in step 1, i.e. $(M_{\boldsymbol{Z}_{\boldsymbol{2}}},M_{X},M_{Y})\perp Y | \left( \boldsymbol{Z}_{\boldsymbol{1}}\boldsymbol{,}\boldsymbol{Z}_{\boldsymbol{2}},X \right)$:

$$P\left( Y=y|X, \boldsymbol{Z}_{\boldsymbol{1}}\boldsymbol{,}\boldsymbol{Z}_{\boldsymbol{2}} \right)= P\left( Y=y|X, \boldsymbol{Z}_{\boldsymbol{1}}\boldsymbol{,}\boldsymbol{Z}_{\boldsymbol{2}},M_{Y}=0,M_{X}=0,M_{Z_{2}}=0 \right)$$

Therefore, the target estimand in the example (i.e. the coefficient for maternal mental illness in the regression model for child behavior), assuming the m-DAG presented in Figure 2a, is recoverable. Not only that, but the above expression shows that a complete records analysis (CRA) will provide consistent estimation for this parameter.

**References**

1. Thoemmes F, Mohan K. Graphical Representation of Missing Data Problems. *Struct Equ Modeling* 2015; **22**: 631-42.

2. Pearl J. *Causality*. New York: Cambridge University Press; 2000.

3. Textor J, van der Zander B. dagitty: Graphical Analysis of Structural Causal Models. 2016 <https://cran.r-project.org/package=dagitty> (5 January 2023, date last accessed).
